# Polymorphism A118G of opioid receptor mu 1 (*OPRM1*) is associated with emergence of suicidal ideation at antidepressant onset in a large naturalistic cohort of depressed outpatients

**DOI:** 10.1038/s41598-019-39622-3

**Published:** 2019-02-22

**Authors:** B. Nobile, N. Ramoz, I. Jaussent, Ph Gorwood, E. Olié, J. Lopez Castroman, S. Guillaume, Ph Courtet

**Affiliations:** 1 Department of Emergency Psychiatry and Post-Acute Care, CHU Montpellier, Montpellier, France; 2 INSERM UMRS1266, Institute of Psychiatry and Neuroscience of Paris, Université Sorbonne Paris Cité, Paris, France; 3 INSERM, U1061, Neuropsychiatry, University Montpellier, Montpellier, France; 4 FondaMental Foundation, Montpellier, France; 5 Department of Psychiatry, CHU Nimes, Nimes, France

## Abstract

Antidepressants have been the object of an international controversy for about thirty years. Some patients are inclined to develop suicidal ideation (SI) at antidepressant onset; this phenomenon is known as Treatment Emergent Suicidal Ideation (TESI), and it has conducted regulatory bodies to prompt warnings on antidepressants. Since, few studies have explored the pharmacogenomics of TESI. Given the growing body of evidence connecting the opioidergic system with suicidal behavior (particularly mu opioid receptor (MOR)), we decided to examine the relationship between two genetic polymorphisms (SNPs) in the opioidergic system and TESI in a sample of 3566 adult depressed outpatients. General practitioners and psychiatrists throughout France followed participants for 6 weeks after an initial prescription of tianeptine, an antidepressant treatment with mu agonism. Suicidal ideation was assessed with the item 10 of the Montgomery-Asberg Depression Rating Scale (item dedicated to SI) at baseline, and after 2 weeks, 4 weeks and 6 weeks. We analysed rs1799971 from the *OPRM1* gene and rs105660 from the *OPRK1* gene. Within the sample, 112 patients reported TESI while 384 did not. We found a significant association between AA genotype of rs1799971 and TESI even after adjustment for potential cofounders (OR = 1.93, 95% CI = [1.07; 3.49]; *p-value* = 0.03). On the other hand there were no significant association between rs1799971 and rs105560 with worsening of suicidal ideation or lifetime suicide attempts. Nevertheless, our results suggest a possible involvement of opioidergic system in TESI.

## Introduction

With more than 800 000 suicide worldwide each year and 20 to 30 more suicide attempts (SA), suicide is a major public health problem^1,2^. The leading cause of suicide and suicide attempts is depression^3^. Indeed, 40 to 80% of suicide attempts are directly linked to depressive episodes^4^ and suicide rates range from 5 to 20% among depressed patients^5^. Antidepressants are the best treatment for severe depressed patients but they are questioned by a controversy. Yet, since 1990 an international controversy began about antidepressants use and treatment emergence or worsening of suicidal ideation (TESI and TWOSI) especially after a publication which described 6 patients who developed suicidal behavior (SB) under fluoxetine^6^. This controversy conducts regulatory bodies to prompt warning about antidepressants use. Finding biomarkers associated to TESI and TWOSI may help to prevent suicidal acts among depressed patients.

So far we know little about the physiopathological mechanisms leading to suicidal ideation (SI) during antidepressant treatment. For one reason, TESI and TWOSI appear only in 10 to 20% of clinical samples^7^, mostly during the first 5 weeks of treatment^8,9^. Some studies have identified socio-demographic and clinical risk factors associated to TESI and TWOSI (i.e. pre-adult onset of depression, gender, depression severity, physical pain…)^3,7,8,10–12^. Several large naturalistic studies investigated candidate genes. They reported associations with genes involved in the neurotrophic and synaptic plasticity systems (*BDNF*, *NTRK2*, and *CREB1*)^13–15^, noradrenergic system (ADRA2A)^14^, glutamatergic system (GRIA3, GRIK2 and GDA)^16–18^, the stress and inflammatory responses (FKBP5 and IL28RA)^19,20^, and the synthesis of glycoproteins (PAPLN)^20^. Those genes have also been implicated in SB or related phenotypes. Based on the few numbers of studies, it is difficult to draw firm scientific conclusions to date. Despite the very large cohort samples, the main methodological issues are the modest size of those studies them, due to the rarity of this phenomenon (10 to 20% according to different studies) and the variability in defining TESI. Four GWAS from these cohorts have been published but again, they investigated relatively small samples that lacked the power to detect effects other than major gene effects^17,18,20,21^.

On the other hand, there is growing evidence suggesting an implication of opioidergic system in the physiopathology of SB. Patients using high doses of opioids seem to be more inclined to have SI and attempt suicide^22–25^. Others studies found low levels of endorphin and mu opioid receptor (MOR) activation in patients presenting a major depressive episode (MDE)^26,27^. In the same vein, post-mortem studies found an increase density of MOR in prefrontal cortex and nucleus caudate in brains of suicide victims, probably as a consequence of a compensatory mechanism^28,29^. Moreover, a recent randomized control trial with suicidal patients demonstrated that very low doses of buprenorphine (a partial mu agonist and kappa antagonist) decreased significantly SI compared to placebo^30^. It has been hypothesized that buprenorphine has an “anti-suicidal” effect by its mu agonism and an antidepressant effect by its kappa antagonism^31^. Another recent randomized controlled trial showed that receiving buprenorphine conducted to a significant decrease of SI in acutely depressed patients with co-morbid opiate addiction^32^. Of note, patients receiving tianeptine were significantly less inclined to develop TWOSI than patients taking antidepressant from other classes^33^. This last study is all the most interesting since that tianeptine is acting on opioidergic system. The results of the latter study show the effect of tianeptine on the opioidergic system. Since they have a similar molecular structure, tianeptine was considered as acting like tricyclic antidepressants until recently. A review article focused on tianeptine action revealed that this molecule had neurobiological properties involved in numerous neurotransmitter systems, on neuronal excitability, neuroprotection and in structural and functional plasticity in many brain regions (amygdala, hippocampus…)^34^. Later studies found that tianeptine was acting also on the glutamatergic system and consequently was considered as a glutamate modulator^35^. Recent research revealed that tianeptine is acting as an agonist of mu opioid receptor (MOR) and that its acute and chronic antidepressant-like behavioral effects come from this mu agonism^36,37^. Moreover, it was demonstrated that the primary metabolite of tianeptine, reproduces the behavioral effects of tianeptine in a MOR-dependent fashion^37^. To our knowledge, no study has yet examined the link between TESI and the opioidergic system. Even if the presence of SI is an important risk factor for any suicidal act, suicide attempts and SI may have different biological background. Indeed, there are more and more studies concerning the ideation-to-action framework, which consider that the development of SI and the progression from ideation to SA are distinct phenomena^38^. This work conducts to the elaboration of three theories of suicide which are the interpersonal theory, the integrated motivational-volitional model, and the three-step theory^39–41^. Nevertheless, given the link between SB, SI and the opioidergic system, it seems possible to suppose a relation between this system and TESI/TWOSI.

In this paper we aim to analyze the effect of two Single Nucleotide Polymorphisms (SNPs) from the opioidergic system on TESI and TWOSI: SNP rs1799971 (A118G) from the first exon of the gene *OPRM1* and rs105660 (36 G > T) from the gene *OPRK1*. The G allele of rs1799971 results on an amino acid change (N40D) that decreases MOR expression and has been associated with an increased sensitivity to social rejection, a higher risk to develop major depressive episode (MDE) after an adverse life event and completed suicide^42,43^. Secondly, rs105660 has been associated with opiate addiction and seems to be a functional polymorphism. Indeed, T allele was significantly associated with opiate addiction^44^. To our knowledge there are no studies about *OPRK1* polymorphisms and MDE or suicidal behavior. In this study, we aimed to analyze association between those SNPs and: 1) TESI, 2) TWOSI and 3) History of SA within a large population of outpatients with MDE followed during 6 weeks by general practitioner or psychiatrist, for whom a treatment by tianeptine was initiated.

## Results

### Patients’ characteristics for TESI

The study sample of TESI group at baseline consisted of 496 patients with a mean age of 48.05 years (SD = 14.75) of whom 38.7% were male.

### SNPs association with TESI

One hundred and twelve patients met the criteria for TESI group and 384 for non-TESI group. Table 1 presents the socio-demographic and clinical data for both groups. Patients from TESI group were significantly more likely to be men (*p* = 0.01), have a lifetime SA (*p* = 0.04), have benzodiazepines coprescription (*p* = 0.01), have an alcohol abuse (*p* = 0.02) and have a changing treatment (*p* = 0.05). Subsequent analyses were adjusted for these factors.

**Table 1:** Association between sociodemographic and clinical data and TESI.

Variables	TESI				P-value
	No		Yes		
	N = 384		N = 112		
	n	%	n	%	
Gender					**0.01**
Men	137	35.7	55	49.1	
Women	247	64.3	57	50.9	
Age (years)	47.84 (14.85)		48.76 (14.47)		0.56
Marital Status					0.95
Single	75	19.6	20	17.9	
Married	232	60.7	68	60.7	
Divorced	54	14.1	18	16.1	
Widower	21	5.5	6	5.4	
Study level					0.22
Under bachelor	144	38	52	47.3	
Bachelor	104	27.4	26	23.6	
Superior Study	131	34.6	32	29.1	
Professional activity					0.47
Working	232	61.4	64	57.7	
Unemployment	21	5.6	10	9	
Retired	71	18.8	18	16.2	
Other	54	14.3	19	17.1	
MDE duration					
<2 months	144	37.5	41	36.6	0.65
[2; 6] months	153	39.8	36	32.1	
>6 months	83	21.6	34	30.4	
Don’t know	4	1	1	0.9	
First MDE					0.12
Yes	240	62.7	61	54.5	
No	143	37.3	51	45.5	
Number of MDE	2.46 (1.84)		2.21 (0.98)		0.91
Age at first MDE (years)	36.38 (15.33)		36.33 (14.18)		0.98
HAD-D Baseline	12.24 (3.88)		12.08 (3.92)		0.69
HAD-A Baseline	12.89 (3.37)		12.61 (3.52)		0.45
HAD total score Baseline	25.13 (6.07)		24.69 (6.08)		0.98
Lifetime suicide attempts					**0.04**
Yes	13	3.5	9	8.3	
No	362	96.5	99	91.7	
Benzodiazepine intake					**0.01**
Yes	166	43.3	62	56.9	
No	217	56.7	47	43.1	
Alcohol abuse					**0.02**
Yes	7	1.8	7	6.6	
No	372	98.2	99	93.4	
Treatment instauration					**0.05**
Yes	330	86.2	87	78.4	
No	53	13.8	24	21.6	

Concerning SNP from *OPRM1*, rs1799971 (A118G), there was a trend for an association between AA genotype and TESI (*p-value* = 0.07; model 0, Table 2), even after adjustments for potential confounders (*p-value* = 0.07; model 1, Table 2). Moreover, when analysed by regrouped genotype there was a significant association between AA genotype and TESI (OR = 1.90, 95% CI = [1.10; 3.30]; *p-value* = 0.02; model 0, Table 2), this association remained significant after adjustments for potential confounders (OR = 1.93, 95% CI = [1.07; 3.49]; *p-value* = 0.03, model 1, Table 2) and even adjustment on depression scores change between baseline and 6 weeks (OR = 2.12, 95% CI = [1.15; 3.93]; *p-value* = 0.02, model 2, Table 2). Allele A was significantly associated with TESI (OR = 1.68, 95% CI = [1.04; 2.72]; *p-value* = 0.04; model 0, Table 3) but this association did not remain significant after adjustment for potential confounders (OR = 1.63, 95% CI = [0.97; 2.72]; *p-value* = 0.07; model 1, Table 3). Interestingly, this association became significant when adjusted on potential confounders and on change in depression scores (OR = 1.82, 95% CI = [1.06; 3.13]; *p-value* = 0.03; model 2, Table 3).

**Table 2:** Association between genotype and TESI.

	TESI				Model 0		Model 1		Model 2	
	No N = 384		Yes N = 112		OR [95%CI]	p-value	OR [95%CI]	p-value	OR [95%CI]	p-value
	n	%	n	%						
**rs1799971**										
GG	12	4	3	3.2	1	0.07	1	0.07		**0.05**
AG	91	30	17	18.1	0.75 [0.19; 2.93]		0.52 [0.12; 2.18]		0.66 [0.13; 3.48]	
AA	200	66	74	78.7	1.48 [0.41; 5.39]		1.09 [0.29; 4.20]		1.46 [0.30; 7.12]	
**rs105660**										
AA	1	0.4	0	0						
AC	31	12.2	15	19.2						
CC	222	87.4	63	80.8						
**rs1799971**										
AG/GG	103	34	20	21.3	1	**0.02**	1	**0.03**	1	**0.02**
AA	200	66	74	78.7	1.90 [1.10; 3.30]		1.93 [1.07; 3.49]		2.12 [1.15; 3.93]	
**rs1799971**										
AA/AG	291	96	91	96.8	1	0.73				
GG	12	4	3	3.2	0.80 [0.22; 2.89]					
**rs105660**										
AA/AC	32	12.6	15	19.2	1	0.14				
CC	222	87.4	63	80.8	0.61 [0.31; 1.19]					
**rs105660**										
AC/CC	253	99.6	78	100						
AA	1	0.4	0	0						

Model 0: Crude association. Model 1: Adjusted on gender, lifetime suicide attempts, treatment instauration, alcohol abuse and benzodiazepine intake. Model 2: Adjusted on gender, lifetime suicide attempts, treatment instauration, alcohol abuse, benzodiazepine intake and change in depression scores.

**Table 3:** Association between allele and TESI.

	TESI				Model 0		Model 1		Model 2	
	No N = 384		Yes N = 112		OR [95%CI]	p-value	OR [95%CI]	p-value	OR [95%CI]	p-value
	n	%	n	%						
**rs1799971**										
G	115	19	23	12.2	1	**0.04**	1	0.07	1	**0.03**
A	491	81	165	87.8	1.68 [1.04; 2.72]		1.63 [0.97; 2.72]		1.82 [1.06; 3.13]	
**rs105660**										
A	33	6.5	15	9.6	1	0.19				
C	475	93.5	141	90.4	0.65 [0.34; 1.24]					

Model 0: Crude association. Model 1: Adjusted on sexe, lifetime suicide attempts, treatment instauration, alcohol abuse and benzodiazepine intake. Model 2: Adjusted on sexe, lifetime suicide attempts, treatment instauration, alcohol abuse, benzodiazepine intake and change in depression scores.

Concerning the rs105660 (36 G > T) SNP of the *OPRK1* gene, analyses in three different genotypes were not possible due to the too small numbers of AA genotype in our sample (Table 2). Even when relying on a genotype-wise analysis, there was no significant association between this SNP and TESI (Table 2, model 0).

### SNPs association with TWOSI, Lifetime SA

The study sample of TWOSI group consisted of 2528 patients with a mean age of 49 years (SD = 14.65), 38.9% were male and the mean HAD baseline was 28.15 (SD = 6.04). Three hundred and nineteen patients (12.6%) met criteria for TWOSI. Concerning lifetime SA, we analysed the whole cohort of patients which consisted of 3566 patients with a mean age of 49.30 years (SD = 14.77), 37.8% were male and the mean HAD baseline was 27.88 (SD = 6.10). Three hundred and twenty-two patients (11.8%) had a lifetime suicide attempts.

None of the SNP was significantly associated with TWOSI (Table 4, model 0) and Lifetime SA (Table 5, model 0).

**Table 4:** Association between genotype and TWOSI.

	TWOSI				Model 0	
	No N = 2209		Yes N = 319		OR [95%CI]	p-value
	n	%	n	%		
**rs1799971**						
GG	42	2.4	4	1.6	1	0.67
AG	453	25.7	69	27.1	1.59 [0.56; 4.60]	
AA	1267	71.9	182	71.4	1.51 [0.53; 4.26]	
**rs105660**						
AA	11	0.8	4	1.8	1	0.27
AC	206	14.1	34	15.4	0.45 [0.14; 1.51]	
CC	1243	85.1	183	82.8	0.41 [0.13; 1.28]	

Model 0: Crude association.

**Table 5:** Association between lifetime history of suicide attempts and genotype.

	Lifetime history of SA				Model 0	
	No N = 2405		Yes N = 322		OR [95%CI]	p-value
	n	%	n	%		
**rs1799971**						
GG	60	2.5	11	3.4	1	0.52
AG	611	25.4	76	23.6	1.47 [0.74; 2.93]	
AA	1734	72.1	235	73	1.35 [0.70; 2.61]	
**rs105660**						
AA	16	0.8	4	1.5	1	0.49
AC	299	15	44	16.1	1.70 [0.54; 5.31]	
CC	1683	84.2	226	82.5	1.86 [0.62; 5.62]	

Model 0: Crude association.

## Discussion

To our knowledge, this study is the first to assess the association between polymorphisms from the opioidergic system and TESI in a large sample of outpatients with MDE. We found that SNP rs1799971 (A118G) from *OPRM1* was significantly associated with TESI, while it was neither associated with the lifetime history of SA or with TWOSI. It is interesting to note that this association remain significant even when adjusted on change in depression score suggesting a potential link between this SNP and TESI independently of remission of depression. Surprisingly, it was the AA genotype which was associated with TESI. In previous studies, the minor allele (G) was associated with a higher sensitivity to social exclusion, social adversity and physical pain^42,45,46^. Moreover, this allele is associated with a significant decrease of MOR protein in brain^47^. Two main hypotheses can be formulated concerning our results. First, the effect of treatments with opioidergic action could partly explain this contradiction. Prior studies in anaesthesia found that G carriers of this SNP needed higher doses of morphine to have the same analgesic effect than A carriers^48,49^ but these results were contradicted by later studies^50,51^. Pharmacogenetic research on patients with alcohol use disorders under naltrexone (a mu antagonist) showed that G carriers responded better to treatment than A carriers^52^. Analogously, G carriers in our study receiving the mu agonist tianeptine^47^ were less inclined to develop TESI than A carriers. Thus, we can hypothesize that G carriers are more sensitive to medication acting on MOR than A carriers. The second hypothesis concerns the hypothalamic-pituitary-adrenocortical (HPA) axis. Indeed, this axis is known to be dysregulated in SB with an increase in its activation among suicidal patients^53^. Since this axis is regulated by the opioidergic system^54^ a MOR antagonist would increase HPA axis activation^55^ while a MOR agonist would modulate this activation^56^. A recent genetic study found that G carriers were less reactive to stress (less activation of HPA axis) compared to A carriers, and this effect was more pronounced in women compared to men^57^. By receiving tianeptine, G carriers could have less activation of their HPA axis and also less SI. Finally, decreased TESI in G carriers may be due to a combination of both hypotheses. Indeed, the receptor variant may be associated with a modification of opioid signaling at the cellular level, qlthought further studies are needed to understand this phenomenon.

Interestingly, we did not find any association between SNP rs105660 (36 G > T) from *OPRK1* and TESI or others outcomes of our study. This is certainly due to the too small numbers of patients with the AA or AC genotype in our samples.

There are some limitations in this study. The first one is the small size of our TESI group, despite the large sample size of the origin cohort, due to the rarity of this phenomenon. Furthermore, prevalence of TESI in our sample is about 3% which is lower than those reported in literature (10–20%)^14^. This small size could be explained by three main hypotheses. Firstly, GENESE is a naturalistic cohort with MDE outpatients, which possibly excludes hospitalized patients with the most severe depression symptoms, perhaps more inclined to develop TESI. Secondly, the small number of TESI patients could be linked to the specific antidepressant class of tianeptine. Indeed, patients taking tianeptine are less inclined to develop TWOSI than patients taking others antidepressants^33^. In that way, it seems logical that treatment received could have an impact on developing TESI or not too. By taking tianeptine, our patients may be consequently less inclined to develop TESI, reinforcing the hypothesis of the possible involvement of opioidergic system within TESI. Finally, there is no consensus on definition of TESI. In this study, we choose to favor specificity. Finally, we had not enough patients with AA or AC genotype of rs105660 from *OPRK1* which made the analyzes inconclusive.

In conclusion, we found a significant association between A118G polymorphism from *OPRM1* and TESI in patients treated with tianeptine. This finding supports the involvement of the opioidergic system in the physiopathology of suicidal behavior. If those findings are replicated it could change clinical practice when introducing an antidepressant treatment. Indeed, we can imagine a systematic genotyping of patients and consequently an adaption of their treatment according to their genotype. More studies are needed to confirm the results and understand the mechanisms underlying the implication of the opioidergic system in SB. This avenue of research could eventually lead to new therapeutics targets or personalized care.

## Material and Methods

### Participants and clinical assessment

GENESE is a large, prospective, naturalistic cohort of 3566 French outpatients diagnosed with MDE and treated with tianeptine. Dosage of tianeptine was chosen by a general practitioner (GP) and ranged between 12.5 and 37.5 mg/d, according to prescription recommendations. Patients were followed for at least 6 weeks between the first and the second visit by the same practitioner. At the first visit, GPs or psychiatrists validated the diagnosis of MDE according to DSM-IV criteria. Demographic data, major depressive disorder history, lifetime SA and alcohol or substance dependence was collected by GPs or psychiatrists at the first visit. Non-inclusion criteria were: age under 18 years old, non-Caucasian ethnicity, alcohol and substance dependence, or any other psychiatric pathology from axis I other than current MDE. The study was performed according to French regulatory guidelines and current codes of Good Clinical Practice. Each patient was informed about the aims and procedures of the study and provided written, signed consent. The study protocol was submitted to and approved by local independent ethics committees (Comité de Protection des Personnes CPP Ile de France XI (CPPIDF11), Centre Hospitalier Intercommunal CHI Poissy Saint-Germain, Saint Germain en Laye, reference no. 08042).

Concerning clinical assessment our priority was to collect self-reported and repeated measures of SI and depression longitudinally (even if we also collected measures of SI realized by practitioners). Patients are more likely to disclose SI in self-reported measures than to a clinician^58^. Depression severity was assessed with a French version of the Hospital Anxiety and Depression Scale (HADS) at baseline, week 2, 4 and 6 by patients. This scale demonstrated a good performance assessing depression severity in both psychiatric and primary care patients and a good change-sensitivity^59^. Most factor analyses found a two-factor solution in accordance with the Anxiety (HADS-A) and Depression (HADS-D) subscales. This scale was chosen for its simplicity of use and understanding and for its good psychometric properties, which have been demonstrated also in outpatient groups^60^.

The major dependent variable was SI, a continuous measure obtained longitudinally along the study as recommended in the consensus statement^58^. Moreover, for this study we needed intermediary evaluation. Because no specific scale has been univocally proposed for clinical practice or for clinical research, we chose to assess SI by using the suicidal item of the self-rated Montgomery–Asberg Depression Rating Scale (MADRS) (item number 10) completed by patients at baseline, week 1, 2 and 6 and by practitioners at baseline and at week 6 (in this study, we only used data from self-reported measures of SI). The ratings range from 0 to 6: 0 to 1) enjoys life or takes it as it comes; 2 to 3) weary of life, only fleeting suicidal thoughts; 4 to 5) probably better off dead, suicidal thoughts are common, and suicide is considered as a possible solution, but without specific plans or intention; and 6) explicit plans for suicide when there is an opportunity, active preparations for suicide. A single suicide item from a depression rating scale, either clinician-rated or self-reported, proved to be a valid approach to assess SI when compared with Beck’s scale^61^. This method was used in large clinical studies such as the STAR*D^12^ and also in more recent studies^62^.

### Single Nucleotide Polymorphisms (SNPs) selection

SNPs selection was made according to 2 criteria: 1) SNPs of opioidergic system that had been previously reported as associated either with suicide, depression or addiction, 2) minor allele frequencies above 5%. As said previously, two SNPs were chosen: rs1799971 from the gene *OPRM1* and rs105660 from the gene *OPRK1*. Hardy-Weinberg equilibrium was respected.

### Genotyping

DNA was collected at baseline by buccal swab. Genotyping was performed using a 5’ exonuclease assay (Taqman, Life Technologies). Assay products were run on an applied Biosystem 7900HT Fast Real-Time PCR System (Life Technologies).

### Phenotype definitions of TESI and TWOSI

Only self-administered questionnaires were used for analyzes. TESI was defined by scoring 0 or 1 at MADRS item score corresponding to SI (MADRS-SI) at baseline and then superior or equal to two at least once during the follow-up (baseline, week 1, 2, 6). Non-TESI patients were scoring 0 or 1 during all the follow-up (baseline, week 1, 2 and 6). SI worsening was defined by an increase of at least one point in the MADRS-SI during the follow-up in comparison to baseline.

### Statistics

Categorical variables were presented as percentages, and quantitative variables as means with standard deviation (SD). Demographic and clinical characteristics between non TESI and TESI patients were analyzed using a univariate logistic regression model. To study the association between genotype data and the groups of patients, logistic regression models were used to estimate the odds-ratios (OR) and their 95% confidence interval (95% CI). Baseline sociodemographic and clinical variables associated with TESI at p < 0.10 were included in the logistic regression models to estimate the adjusted OR and 95%CI. The same methodology was used for TWOSI and lifetime SA.

The significance level was set at *P* < 0.05. Analyses were performed using the SPSS statistical software (version 23.0.0.2; IBM SPSS Statistics for Windows. Armonk, NY: IBM Corp).
